# European Correspondence

**Published:** 1877-03

**Authors:** W. S. Kendrick

**Affiliations:** Paris


### EUROPEAN CORRESPONDENCE.

PARIS, October, 1876.

*Dr. W. F. Westmoreland:*

DEAR SIR—During the month of August I wrote you a long letter from London, giving you some little idea of that city and its hospitals.

While many of the men who figured so conspicuously in the medical arena when you were here, long since have been gathered to their fathers, yet, prompted by the feeling that a few lines, however unimportant they may be, from the scenes of your former labors, might possibly interest you, I send the following.

Dr. Scott and I, laboring together for several months in London, separated early in August; he going to Berlin, and I in a short time to this city. I had a pleasant ride from London to Paris; without a day's recreation since reaching Europe, I was delighted to get, for even a few hours, out of the great noisy city of London, and enjoyed very much the sail on the English Channel from Newhaven to Dieppe, and the beautiful and attractive country scenery between the latter place and this.

Settling down at once in the Latin Quarter, I was soon busily engaged in visiting the various departments of medicine. I have been here some weeks, and am well pleased with the hospitals, especially some branches. Without going into detail, I thought probably you might be interested in knowing some of the most prominent men now connected with the schools and hospitals; and thus I give you a few, taken from my visits and also from the announcement of the school. The following are connected with the School of Medicine proper, the clinical lecturers are more properly mentioned with the hospitals: Med. Physics, M. Gavarret; Med. Pathology, M. Ollivier; Anatomy, M. Sappey; General Pathology and Therapeutics, M. Chauffard; Med. Chemistry, M. Wurtz; Surgical Pathology, M. Dalbeau; Histology, M. Robin. These are the most important; a few being omitted. The principal clinical lecturers at the various hospitals are the following: Clinical Medicine, M. Lu, Hotel Dieu; M. Lasigne, Pitie; M. Hardy, Charity; and M. Petain at the Necker. Clinical Surgery, M. Gosselin, at the Charity; M. Richet, Hotel Dieu; M. Broca, Hospital of Cliniques; and M. Verneuil at the Pitie. M. Depaul, Clinical Obstetrical Teacher; M. Blochez, Diseases of Children, and quite a number of others, whose names I will not weary you with. Of course there are others connected with the hospitals who enjoy quite a reputation, and who also hold clinical lectures. Prominent among them is M. Pean, one of the most distinguished men of Paris, and surgeon at the Saint Louis. I believe he is considered the Spencer Wells of France on ovariotomy. Yet I hardly think he operates with the same ease and facility as that illustrious man of London. Besides his operations for ovariotomy, they tell me here that he has removed the spleen twice successfully, and also amputated the fundus uteri ten or twelve times, with a mortality of only twenty per cent.

M. Tilleaux also is considered one of the prominent surgeons, and is probably the leading man at the Lariboisiere. I have seen him operate quite a number of times, and have been much pleased.

I may be mistaken, but am inclined to think that Broca exjoys an equal, if not the highest, reputation among French physicians of the present day, especially in certain departments.

While I have not visited all the hospitals, yet have been to the most important, and find among them, the Hotel Dieu, Lariboisiere, Saint Louis, Pitie, and the hospital opposite the School of Medicine, among the most interesting. I have also had an opportunity of visiting the large Museums of Anatomy and Surgery connected with the various hospitals and schools; and while they contain many things of interest, and all that are necessary, yet they are not so large as I expected to find. The out-patient departments are well attended, and afford considerable advantages to those who desire to attend them. I am specially well pleased with the obstetrical wards in the hospital of the Cliniques. There are two wards devoted entirely to this branch, containing quite a number of beds, which are nearly all the while filled with pregnant women. The professor visits these wards every morning, and the students have all the advantages they could desire, both in teaching and in examinations. All the births that take place during the day can be seen in a room specially prepared for that purpose. The forceps are often applied in the presence of the class, and all the details of labor fully described and taught.

They have instituted here recently a procedure in obstetrics which they claim to be original and new, viz: by external manipulation the presentation of the fœtus is determined a short time before full term, and if any portion is presenting besides the head, and most especially if placenta prævia, the fœtus is at once turned, which is readily done by external means alone; the head is forced down as the presenting part, and held in that position by lacing the abdominal walls.

In speaking of the general hospitals, a new building, opposite the old one, is about completed for the Hotel Dieu. This will be one of the finest hospital buildings in Paris when fully arranged and furnished. Besides these leading hospitals, all the specialties are to be found in others. The hospitals and clinics for diseases of the eye are numerous; some seven or eight being located but a few minutes walk from each other. The most prominent and largest clinics are probably to be found at DeWecker's, Gale Zaskis, and Desmarres, though some of the others I have not attended.

This is one branch which in Paris is taught very well; and while the large number of patients is not to be found here that daily visit Moorefield's in London, yet a sufficient quantity for all practical purposes can be always seen. Certain days are devoted at each clinic to special subjects, viz: one or two days of each week to external diseases of the eye; another principally to ophthalmology, etc. Lectures are also delivered usually several times a week on some special subject. I think these clinics afford to the student a very good opportunity of pursuing his studies in ophthalmology.

I have seen nothing very special or new in the treatment of diseases of the eye from that practiced in our own country. The mode of operating for cataract and the treatment of external diseases are about the same as there. I find one or two here, and also in London, who nearly always make their iridectomies below instead of above the pupil. I saw practiced at the Royal Ophthalmic, at London (Moorefield's), by Bowman and Critchett, two different operations on the same patient. In cataract of both eyes, the incision was made in one in the corneo-scleral junction, and in the other entirely in the cornea. The former was usually successful, while sloughing of the cornea often resulted in the latter.

There is but one special clinic for diseases of the ear, and also another general, held once a week at the Hospital Lariboisiere. The special is conducted by M. Garrigan-Desarenes, and the general by M. Tilleaux.

Besides the department in the general hospitals for the urinary organs, there is also a special clinic held by M. Mallez, where ample practice can be seen. The principal opportunity for diseases of the skin is at the Saint Louis, where special attention is given to that subject. There are also hospitals devoted to diseases of children; and again for syphilitic men and women. The latter I have not attended; while not by any means master of the subject, yet such fine opportunities were found in London, where so much of it exists, I have given that subject but little attention here. The lowest classes of London—I mean the debased, degraded classes—are certainly among the lowest in the world, and one can find them pouring into the syphilitic hospitals in large numbers almost every day.

I have been here some weeks, and have seen quite a number of surgical operations. The surgery is similar to that practiced by yourself and other surgeons of America, and also in other places of Europe, and I now remember nothing specially new that would interest you. Each surgeon here, as elsewhere, has some minor peculiarities about operating, but in the end they do not materially differ. Some, in amputating, make the circular, while others the flap operation. Have seen again, instead of the flaps being made above and below, the arc cut laterally, a long and a short one, and brought together on the side. Some close the flaps with sutures, while others do not, allowing them, as is practiced in New York to some extent, to heal by granulations. The almost universal mode of dressing wounds in London is carbolic acid applied in some form—usually carbolized oil or water on either lint or cotton-wool. Some surgeons there use chloride of zinc, and others what they call lotion plumbi. Here carbolic acid is also extensively used, especially for washing wounds; and afterwards they are often dressed with a solution of either chloride of zinc, alcohol, or glycerine in water. Offensive, suppurating wounds are said by some English surgeons to do better dressed with chloride of zinc than with anything else. Lint, cottonwool, linen, etc., etc., are largely used in London for dressing wounds, but charpie is almost universally applied directly to the wound here, though the principle of the two is the same. They use enormous quantities of cotton-wool in putting up their wounds after amputations, injuries, etc., claiming that better results are obtained by this means than by any other. For instance, after an amputation the wound is dressed with charpie or cotton-wool, medicated with whatever they may desire to use, and afterward it is enveloped with the roller of cotton-wool, until the dressing is several inches in thickness; the whole is secured with a roller bandage and left undisturbed for seven or eight days, unless some complications arise causing its removal. They claim this method keeps the stump or wound warm, the muscles quiet, prevents all external injuries, and anything in the atmosphere likely to prove injurious.

I have not seen the carbolic spray (Lister's method) used here as in London, though many of the surgeons there do not employ it. Probably the methods of performing a few of the surgical operations might not be uninteresting to you. The principal for hemorrhoids is the clamp and actual cautery after the tumors have been excised. The same is practiced in London, with the addition of the ligature, and also at times the wire and ecraseur, heated by means of the battery. Both the English and French surgeons are using in many operations where it is appropriate the platinum blade, which is attached to a spray apparatus, and petroleum used for heating. It is fitted into a handle which is hollow; the handle is then connected with the spray apparatus by means of a tube, the blades first heated, and then whatever intensity is desired is afterwards kept up by means of the petroleum spray. You may be familiar with it, though it is claimed in Europe as being entirely new. The operation for hemorrhoids, I forgot to mention, with the chain ecraseur, so extensively used in Paris years ago, has to a great extent been abandoned, though not altogether. In London the principal treatment for stricture of the urethra is by gradual dilatation and division of the stricture by incision. About the same method is practiced here. The instrument used is an American one, but the name has slipped my memory at the present. The excission of joints is not practiced near so extensively here as in London. In the latter place they claim fine success for the operation. The almost universal custom in England, in gynæcological examinations and manipulations, is with the patient on the left side; here, as in America, principally on the back.

I think the French give much less medicine than either the Americans or English, as a rule; though of this I would not speak positively. In speaking of the special hospitals, I failed to mention Fanoel on the throat, who has a very good clinic.

I could go on and on, giving you some little idea of what I have seen since last spring; but will desist, fearing I may weary you. Paris is probably the cheapest place in Europé to study medicine, if no degrees are desired, and not a great deal of private instruction; no charge being made for attending the lectures and clinics. The hospitals of London afford almost the same advantages; small fees being required by some, and none by others, with some additional ones which can hardly be secured here, unless by remaining some length of time, and going up for the degrees. Like all other places, Paris has its advantages and disadvantages; but while you know it has lost the reputation of being the great medical centre of Europe, crushing out all opposition, yet the march onward is still in the front rank, and there are many things to attract the medical man. Private instruction in almost any branch can be obtained, and the teaching is very fine. I have been taking advantage of some of these. This instruction costs in proportion to the time consumed. If one wishes to continue any special course—say operative surgery—for three months, the cost is about 100 francs ($20); if for but one month, 50 francs ($10). Courses on auscultation and percussion, obstetric manipulations, gynæcological operations, microscopy, etc., can be obtained without any trouble, and nearly, if not all of them, at any time of the year. The spacious anatomical rooms, the ample material for dissection, and the able corps of prosectors, afford ample opportunities for those wishing to dissect.

There are some five or six thousand medical students in Paris each year. They are required to attend five years before applying for graduation, though Parisian life is so attractive, I fear many find a good portion of that time in pursuits other than medical. One thing can be said of the French, for kindness, affability and politeness, they are certainly unsurpassed.

I intended mentioning that chloroform is universally used here, and to a large extent in London. I have witnessed several patients on the very verge of the grave from its administration, and which the most active exertions alone saved. Chassaignac, in the zenith of his glory when you were in Paris, is now an old man and retired from the hospital practice. I have visited the beautiful anatomical models which are made nowhere like here; it is quite a treat to spend a few hours among them. Also the instrument houses of Mathieu, Louis and others.

I cannot close this already too long letter without giving vent to at least a few expressions on the city. The day after I reached Paris, I felt as the Queen of Sheba did when on seeing the glory and splendor of Lebanon, said "the half had not been told her." Paris for beauty, grandeur, magnificence and brilliancy is the grand mistress of the world of the present day. It is the modern Rome of this age. It is the pride of France and the glory of her people. London, probably more than twice the size of Paris, a city almost without bounds as it were, contracts her own debts and pays them irrespective of neighboring towns and the surrounding country. The public debts of Paris are the debts of France, or in other words we might say, Paris is France. The Louvre with its endless display of paintings and sculpture, touched and chiselled by the most gifted of those professions, presents attractions to be found nowhere equaled in the world. The garden of Tuilleries, Place de la Concorde, Champs d'Elyssee with their unrivalled beauties and attractive splendors, with their shady, flowery walks, their playing fountains, their fine bands of music paid by the government to enchant daily an admiring populace—with the whirl and dash of the thousands of vehicles that daily fill these broad avenues and royal places can possibly be found nowhere as in Paris. To say nothing of the beautiful houses, the straight and shady boulevardes, the large flower gardens and the shady parks. But I will close. I could write you much of the hospital practice and give you many notes taken from the clinical lectures of such men as Sir James Paget, for the JOURNAL, but do not suppose they would specially interest the medical public.

Truly yours, W. S. KENDRICK.

